# Supplementary material for: Site-specific synergy in heterogeneous single atoms for efficient oxygen evolution
Source: Nat Commun. 2025 Mar 15;16:2573. doi: 10.1038/s41467-025-57864-w (PMC11910543; doi:10.1038/s41467-025-57864-w)
Supplement: Supplementary file 1 — Supplementary Information [file 41467_2025_57864_MOESM1_ESM.pdf]

Supplementary Information for

Site-specific synergy in heterogeneous single atoms for efficient oxygen evolution

Peiyu Ma[1†], Jiawei Xue[1†], Ji Li[1†], Heng Cao[1], Ruyang Wang[1], Ming Zuo[2], Zhirong Zhang[2*], Jun Bao[1,3,4*]

[1]National Synchrotron Radiation Laboratory, University of Science and Technology of China, Hefei, Anhui 230026, P. R. China

[2]Hefei National Research Center for Physical Sciences at the Microscale, University of Science and Technology of China, Hefei, Anhui 230026, P. R. China

[3]Key Laboratory of Precision and Intelligent Chemistry, University of Science and Technology of China, Hefei, Anhui 230026, P. R. China

[4]*i*ChEM (Collaborative Innovation Center of Chemistry for Energy Materials), University of Science and Technology of China, Hefei, Anhui 230026, P. R. China

*Corresponding author E-mail: zzhirong@ustc.edu.cn (Z.Z.); baoj@ustc.edu.cn (J.B.)

[†]These authors contributed equally to this work.

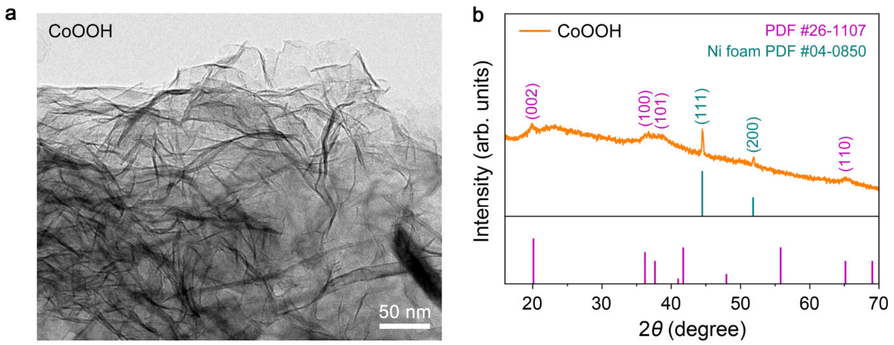

Supplementary Figure 1 | Morphology and structure characterizations of CoOOH. a. TEM image of CoOOH. **b**. XRD pattern of CoOOH. The characteristic peaks that emerged at 2θ = 44.5° and 51.8° were attributed to the (111) and (200) planes of Ni foam, respectively (PDF #04-0850). Source data are provided as a Source Data file.

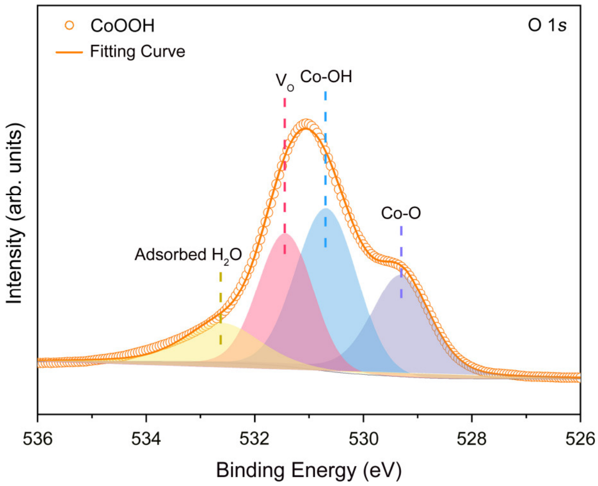

Supplementary Figure 2 | Electronic structure characterization. O 1*s* XPS spectrum of CoOOH. Source data are provided as a Source Data file.

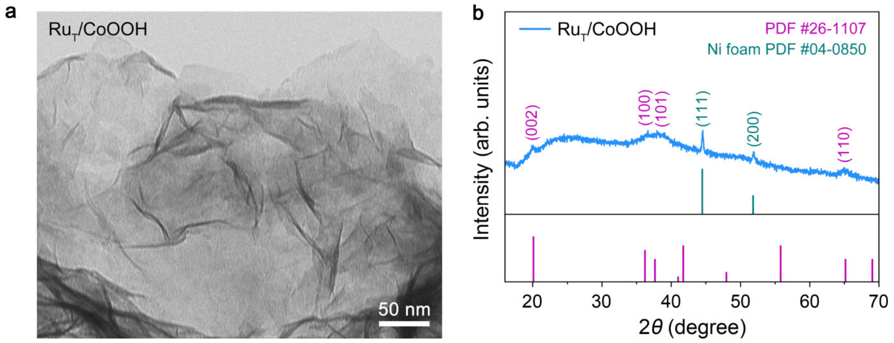

Supplementary Figure 3 | Morphology and structure characterizations of Ru_T/CoOOH. a. TEM image of Ru_T/CoOOH. **b**. XRD pattern of Ru_T/CoOOH. Source data are provided as a Source Data file.

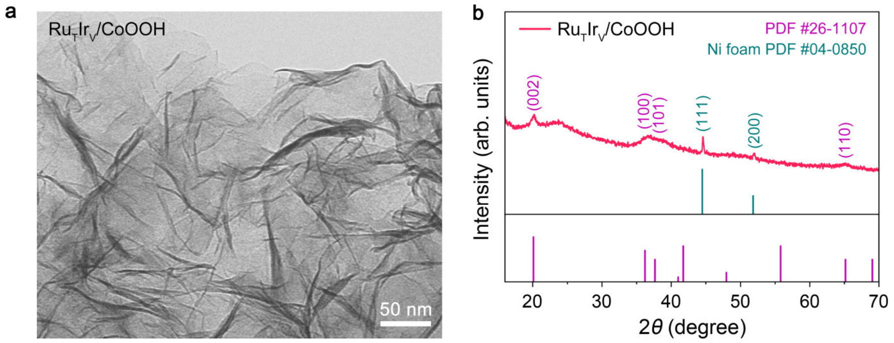

Supplementary Figure 4 | Morphology and structure characterizations of Ru$_T$Ir$_V$/CoOOH. **a**. TEM image of Ru$_T$Ir$_V$/CoOOH. **b**. XRD pattern of Ru$_T$Ir$_V$/CoOOH. Source data are provided as a Source Data file.

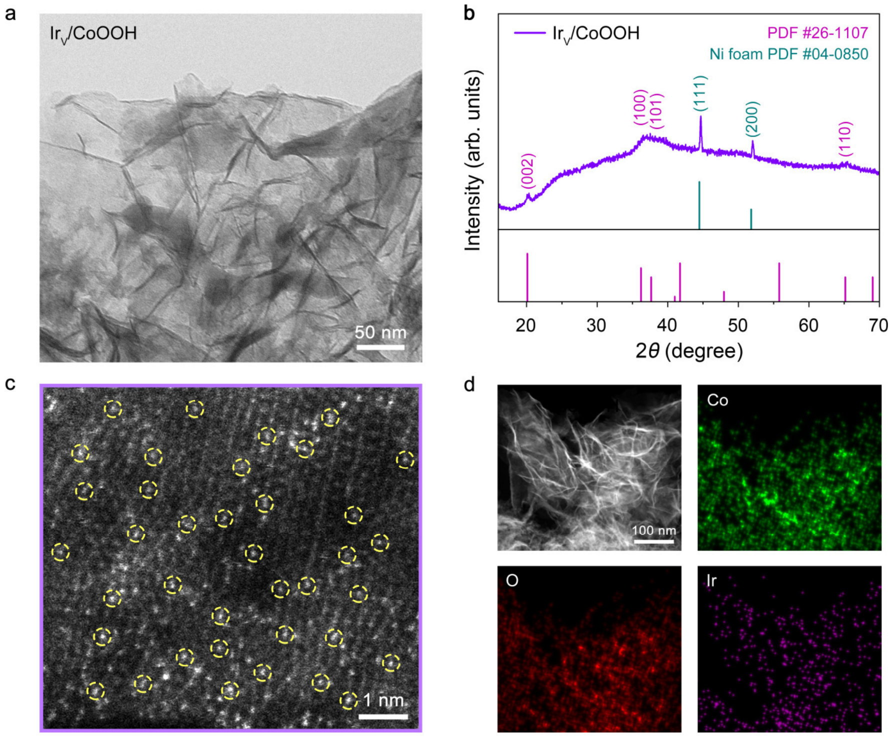

Supplementary Figure 5 | Morphology and structure characterizations of Ir$_V$/CoOOH. a. TEM image. **b**. XRD pattern. **c**. HAADF-STEM image. **d**. EDX elemental mapping. Source data are provided as a Source Data file.

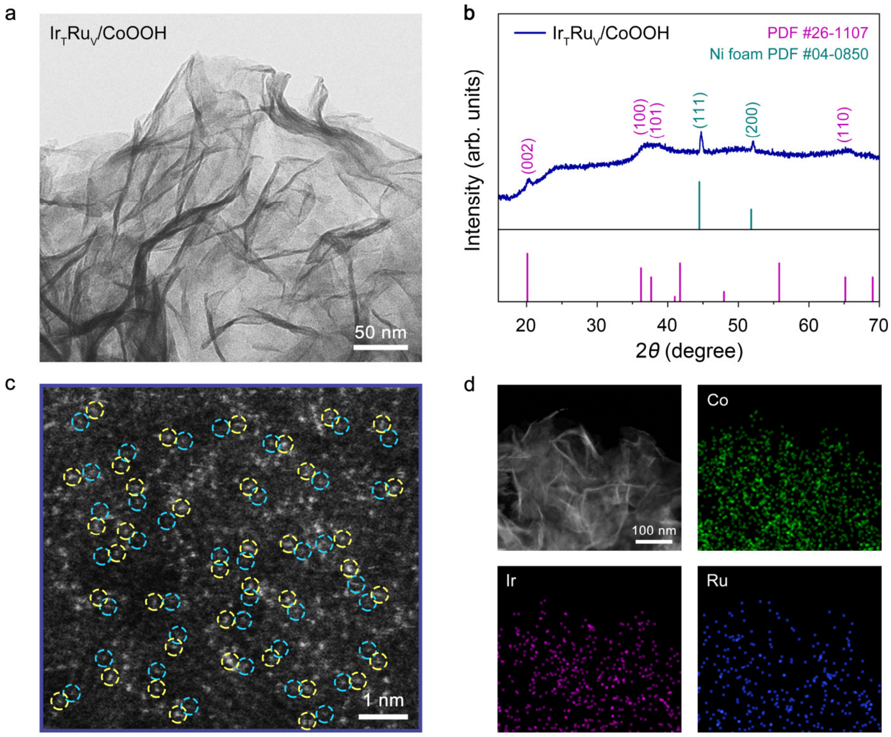

Supplementary Figure 6 | Morphology and structure characterizations of Ir_TRu_V/CoOOH. **a**. TEM image. **b**. XRD pattern. **c**. HAADF-STEM image. **d**. EDX elemental mapping. Source data are provided as a Source Data file.

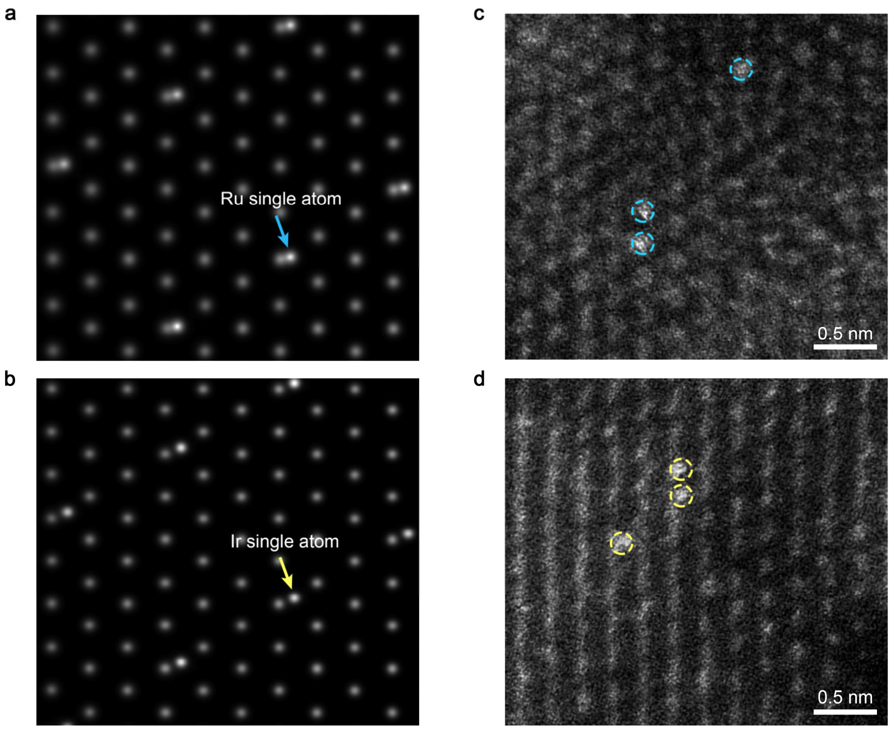

Supplementary Figure 7 | Imaging of the anchoring sites of Ru and Ir single atoms on CoOOH. a. **b**. Simulated HAADF-STEM images of Ru_T/CoOOH (**a**) and lr_VCoOOH (**b**) from [-111] projection. **c**. **d**. Experimental HAADF-STEM images of Ru_T/CoOOH (**c**) and Ir_V/CoOOH (**d**). Ru and Ir single atoms were indicated by blue and yellow circles, respectively.

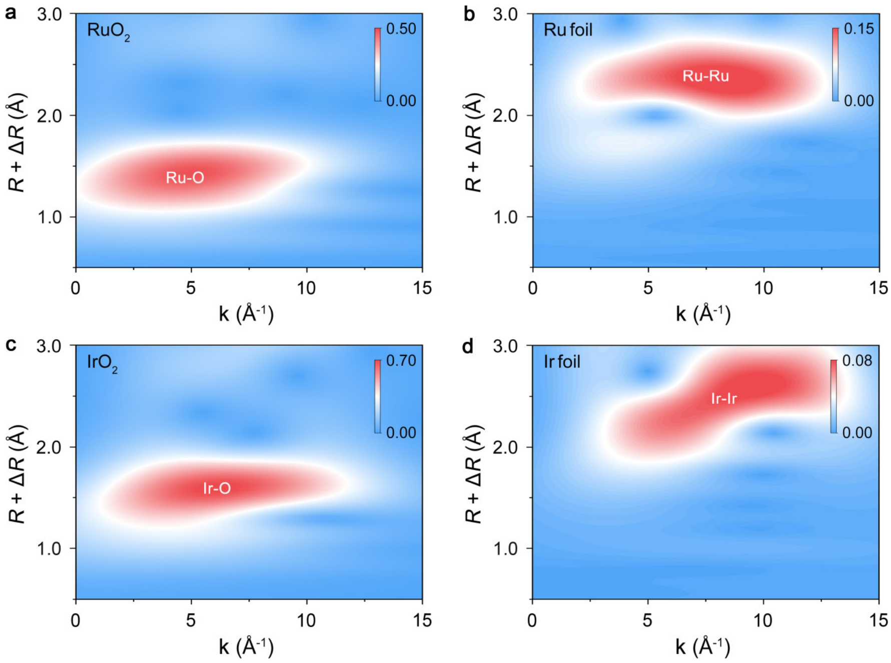

Supplementary Figure 8 | Wavelet transform of Ru *K*-edge and Ir L_3-edge EXAFS oscillations. a. **b**. WT of the $\mathbf{k}^3$-weighted Ru *K*-edge EXAFS signals of RuO_2 (**a**) and Ru foil (**b**). **c**. **d**. WT of the $\mathbf{k}^3$-weighted Ir L_3-edge EXAFS signals of IrO_2 (**c**) and Ir foil (**d**). *R* and **k** denoted radial distance and wave vector, respectively. Source data are provided as a Source Data file.

Supplementary Table 1 | Fitting results of Ru *K*-edge EXAFS spectra for Ru_T/CoOOH and Ru_TIr_V/CoOOH.

Samples	Path	*R* (Å)	*CN*s	σ^2 (10^{-3})	ΔE_0 (eV)	*R*-factor
Ru_T/CoOOH	Ru-O	2.00 ± 0.02	5.0 ± 0.8	5.3	-1.2	0.01
Ru_TIr_V/CoOOH	Ru-O	2.00 ± 0.02	5.0 ± 0.8	5.0	-0.4	0.01

R, the distance between the absorber and backscatter atoms. The *R* value was phase corrected during fitting process; *CN*s, coordination numbers; σ^2, Debye-Waller factors; ΔE_0, the inner potential correction that accounts for the difference in the inner potential between the sample and the references; S_0^2, the amplitude reduction factor, the S_0^2 for the Ru *K*-edge EXAFS spectra fitting was determined to be 0.74; *R*-factor, the goodness of fit.

Supplementary Table 2 | Fitting results of Ir L_3-edge EXAFS spectra for Ir_V/CoOOH and Ru_TIr_V/CoOOH.

Samples	Path	R (Å)	CNs	σ^2 (10^{-3})	ΔE_0 (eV)	R-factor
Ir_V/CoOOH	Ir-O	2.00 ± 0.01	6.1 ± 1.3	10.0	10.0	0.02
Ru_TIr_V/CoOOH	Ir-O	2.00 ± 0.01	6.0 ± 1.1	7.9	10.0	0.02

R, the distance between the absorber and backscatter atoms. The R value was phase corrected during fitting process; CNs, coordination numbers; σ^2, Debye-Waller factors; ΔE_0, the inner potential correction that accounts for the difference in the inner potential between the sample and the references; S_0^2, the amplitude reduction factor, the S_0^2 for the Ir L_3-edge EXAFS spectra fitting was determined to be 1.05; R-factor, the goodness of fit.

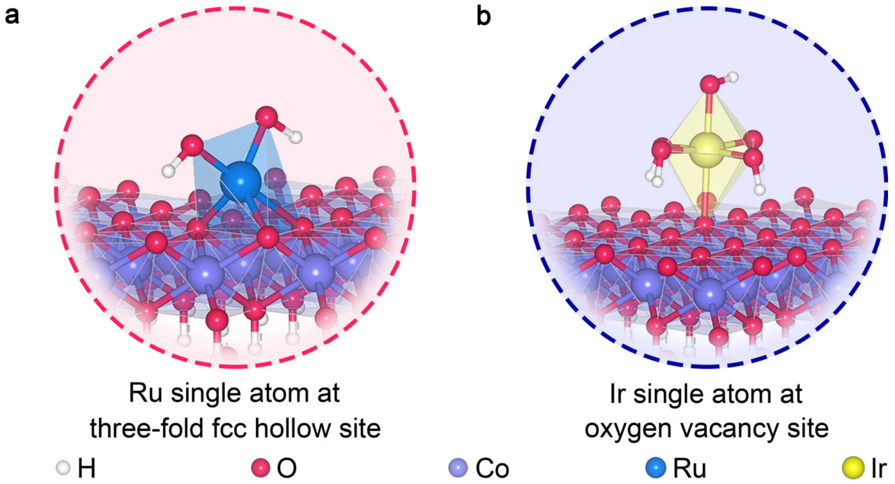

Supplementary Figure 9 | Atomic structural analysis of the Ru and Ir single atoms at diverse sites. a. Structural model of Ru single atoms at three-fold fcc hollow sites. **b**. Structural model of Ir single atoms at V_O sites.

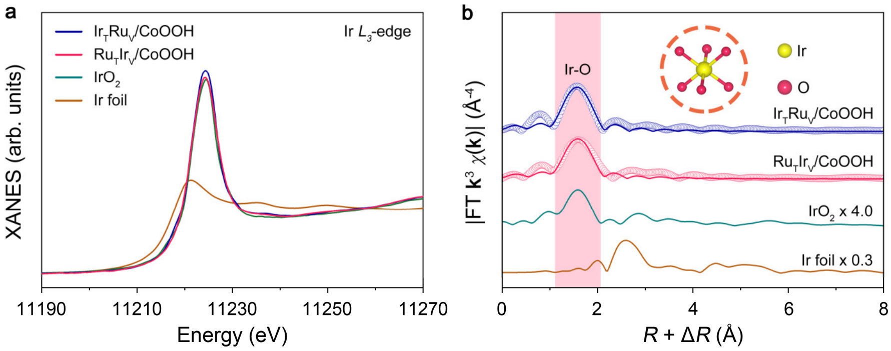

Supplementary Figure 10 | Atomic structural analysis of Ir_TRu_V/CoOOH and Ru_TIr_V/CoOOH. a. XANES spectra at the Ir L_3-edge of Ir_TRu_V/CoOOH and Ru_TIr_V/CoOOH. **b**. Experimental and fitting EXAFS spectra at the Ir L_3-edge of Ir_TRu_V/CoOOH and Ru_TIr_V/CoOOH. R and **k** denoted radial distance and wave vector, respectively. The inset atomic models are the first-shell coordination of Ir single atoms at three-fold fcc hollow sites. The red and yellow spheres represent O and Ir atoms, respectively. Source data are provided as a Source Data file.

Supplementary Table 3 | Fitting results of Ir L_3-edge EXAFS spectra for Ir_TRu_V/CoOOH and Ru_TIr_V/CoOOH.

Samples	Path	*R* (Å)	*CN*s	σ^2 (10^{-3})	ΔE_0 (eV)	*R*-factor
Ir_TRu_V/CoOOH	Ir-O	2.00 ± 0.02	6.0 ± 1.1	6.5	9.8	0.01
Ru_TIr_V/CoOOH	Ir-O	2.00 ± 0.01	6.0 ± 1.1	7.9	10.0	0.02

R, the distance between the absorber and backscatter atoms. The *R* value was phase corrected during fitting process; *CN*s, coordination numbers; σ^2, Debye-Waller factors; ΔE_0, the inner potential correction that accounts for the difference in the inner potential between the sample and the references; S_0^2, the amplitude reduction factor, the S_0^2 for the Ir L_3-edge EXAFS spectra fitting was determined to be 1.05; *R*-factor, the goodness of fit.

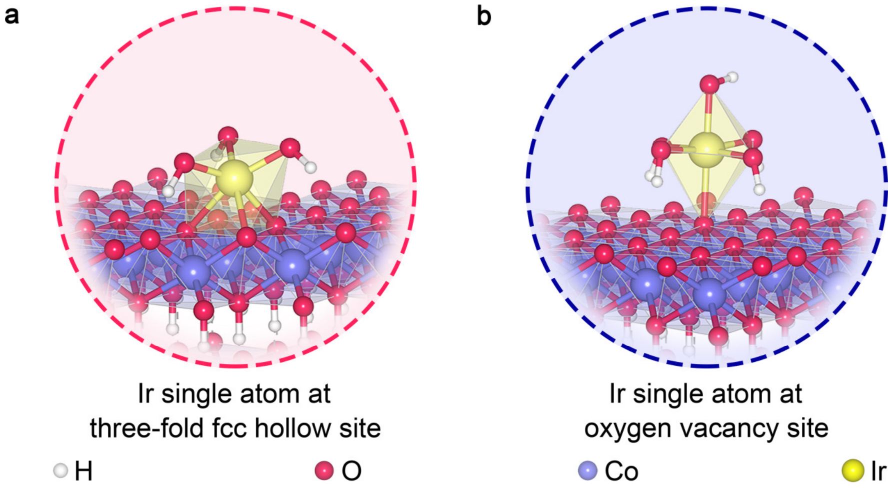

Supplementary Figure 11 | Atomic structural analysis of the Ir single atoms at diverse sites.

a. **b**. Structural model of Ir single atoms at three-fold fcc hollow sites (**a**) and V_O sites (**b**).

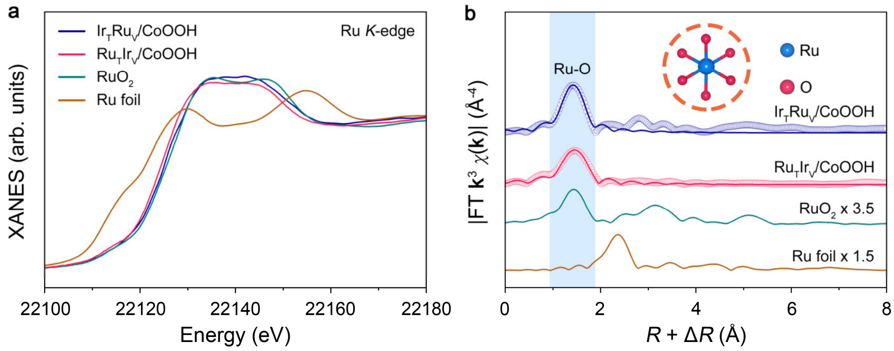

Supplementary Figure 12 | Atomic structural analysis of Ir_TRu_V/CoOOH and Ru_TIr_V/CoOOH. a. XANES spectra at the Ru *K*-edge of Ir_TRu_V/CoOOH and Ru_TIr_V/CoOOH. **b**. Experimental and fitting EXAFS spectra at the Ru *K*-edge of Ir_TRu_V/CoOOH and Ru_TIr_V/CoOOH. *R* and **k** denoted radial distance and wave vector, respectively. The inset atomic models are the first-shell coordination of Ru single atoms at V_O sites. The red and blue spheres represent O and Ru atoms, respectively. Source data are provided as a Source Data file.

Supplementary Table 4 | Fitting results of Ru *K*-edge EXAFS spectra for Ir_TRu_V/CoOOH and Ru_TIr_V/CoOOH.

Samples	Path	*R* (Å)	*CN*s	σ^2 (10^{-3})	ΔE_0 (eV)	*R*-factor
Ir_TRu_V/CoOOH	Ru-O	2.00 ± 0.02	6.1 ± 1.3	3.8	-4.8	0.02
Ru_TIr_V/CoOOH	Ru-O	2.00 ± 0.02	5.0 ± 0.8	5.0	-0.4	0.01

R, the distance between the absorber and backscatter atoms. The *R* value was phase corrected during fitting process; *CN*s, coordination numbers; σ^2, Debye-Waller factors; ΔE_0, the inner potential correction that accounts for the difference in the inner potential between the sample and the references; S_0^2, the amplitude reduction factor, the S_0^2 for the Ru *K*-edge EXAFS spectra fitting was determined to be 0.74; *R*-factor, the goodness of fit.

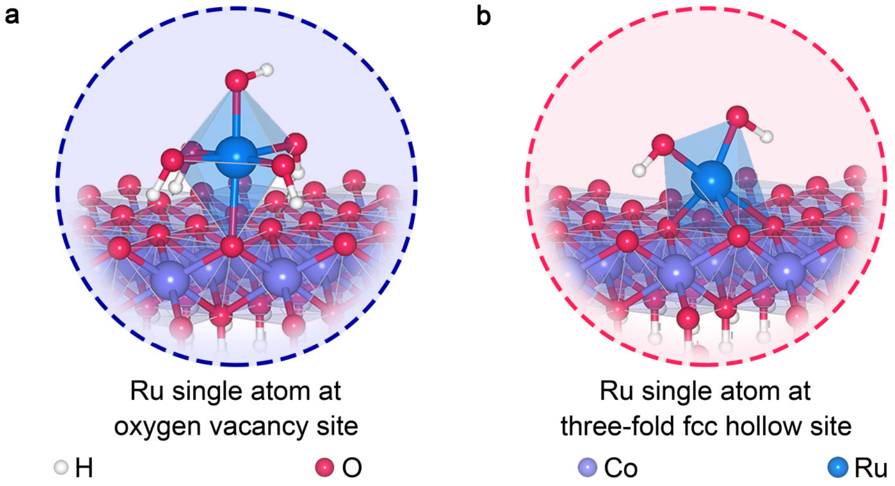

Supplementary Figure 13 | Atomic structural analysis of the Ru single atoms at diverse sites. a. **b**. Structural models of Ru single atoms at V_O sites (**a**) and three-fold fcc hollow sites (**b**).

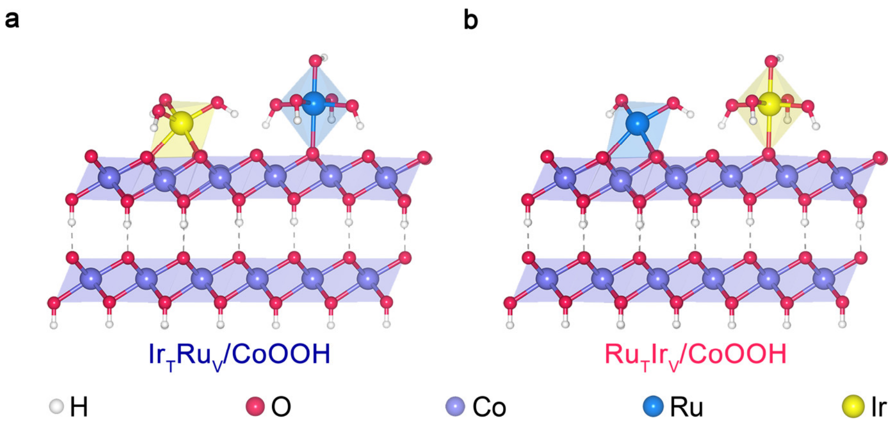

Supplementary Figure 14 | Schematic structural model of the site-specific single-atom catalysts. a. **b**. Structural models of Ir_TRu_V/CoOOH (**a**) and Ru_TIr_V/CoOOH (**b**).

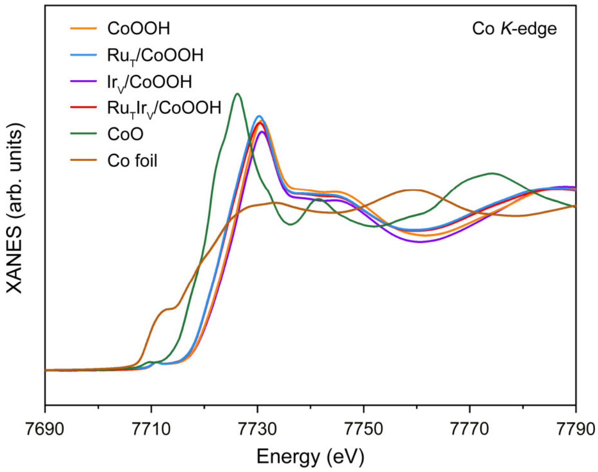

Supplementary Figure 15 | Normalized XANES spectra at the Co *K*-edge of CoOOH, Ru$_T$/CoOOH, Ir$_V$/CoOOH, and Ru$_T$Ir$_V$/CoOOH. Co foil and CoO were used as references. Source data are provided as a Source Data file.

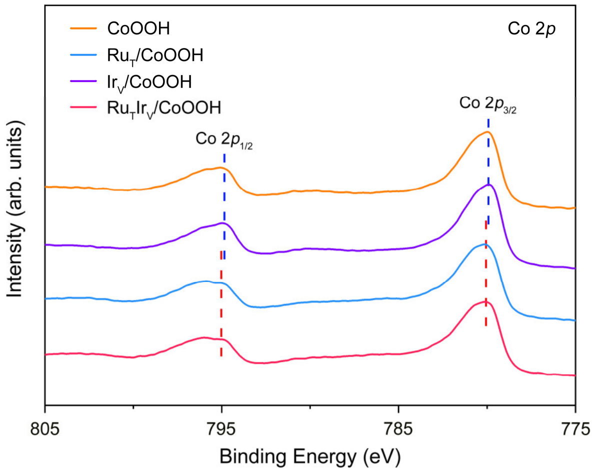

Supplementary Figure 16 | Electronic structure characterization. Co 2*p* XPS spectra of CoOOH, Ru_T/CoOOH, Ir_V/CoOOH, and Ru_TIr_V/CoOOH. Source data are provided as a Source Data file.

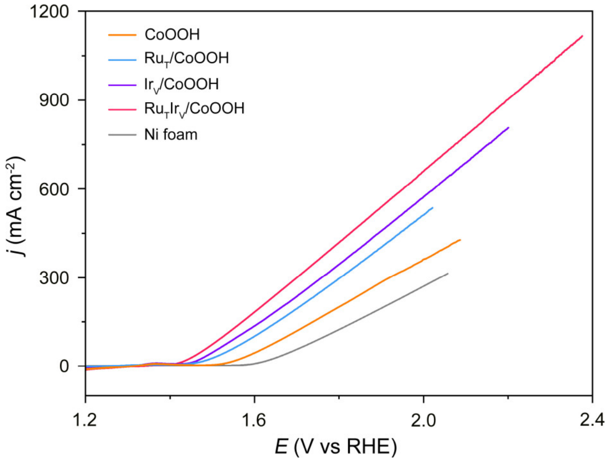

Supplementary Figure 17 | Electrocatalytic performance towards oxygen evolution. Polarization curves of catalysts towards oxygen evolution in 1.0 M KOH electrolyte without *iR*-compensation. Source data are provided as a Source Data file.

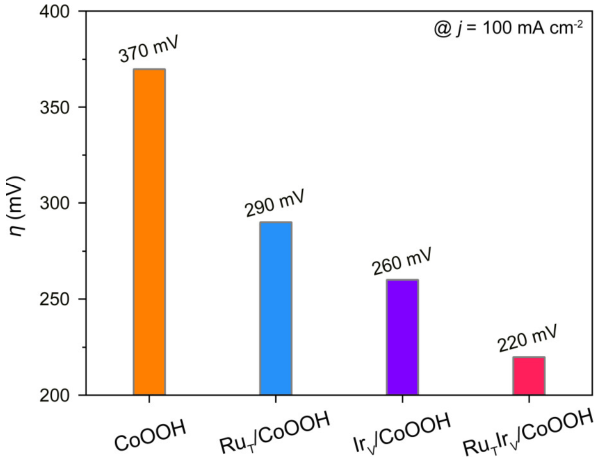

Supplementary Figure 18 | Electrocatalytic performance towards oxygen evolution. Overpotentials of CoOOH, Ru_T/CoOOH, Ir_V/CoOOH, and Ru_TIr_V/CoOOH at a current density of 100 mA cm^{-2}. Source data are provided as a Source Data file.

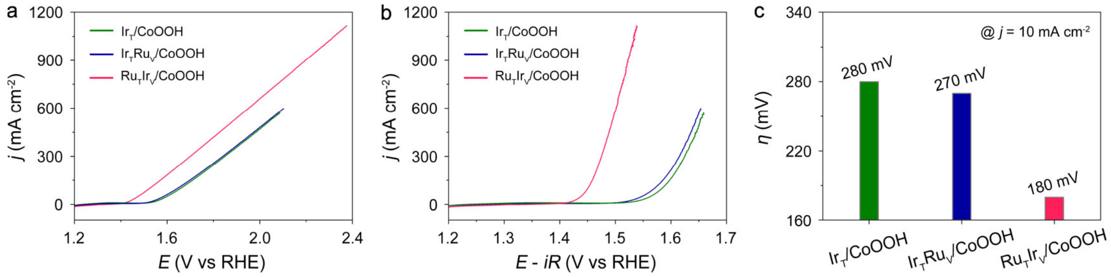

Supplementary Figure 19 | Electrocatalytic performance towards oxygen evolution of site-specific single-atom catalysts. a. Polarization curves of Ir_T/CoOOH, Ir_TRu_V/CoOOH, and Ru_TIr_V/CoOOH towards oxygen evolution in 1.0 M KOH electrolyte without *iR*-compensation. **b**. Polarization curves of Ir_T/CoOOH, Ir_TRu_V/CoOOH, and Ru_TIr_V/CoOOH towards oxygen evolution in 1.0 M KOH electrolyte with *iR*-compensation, *R* was measured to be 0.75 Ω. **c**. Overpotentials of Ir_T/CoOOH, Ir_TRu_V/CoOOH, and Ru_TIr_V/CoOOH at a current density of 10 mA cm^{-2}. Source data are provided as a Source Data file.

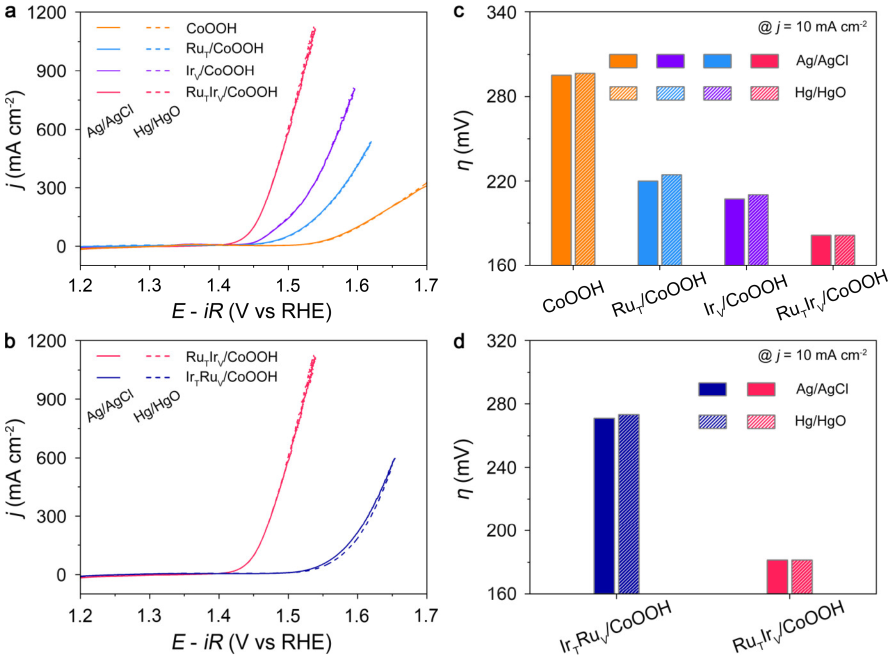

Supplementary Figure 20 | Reproducibility of electrochemical performance. a. **b**. Polarization curves of catalysts towards oxygen evolution in 1.0 M KOH electrolyte using Ag/AgCl and Hg/HgO as the reference electrode, respectively. **c**. **d**. Overpotentials of samples at a current density of 10 mA cm^{-2}. Source data are provided as a Source Data file.

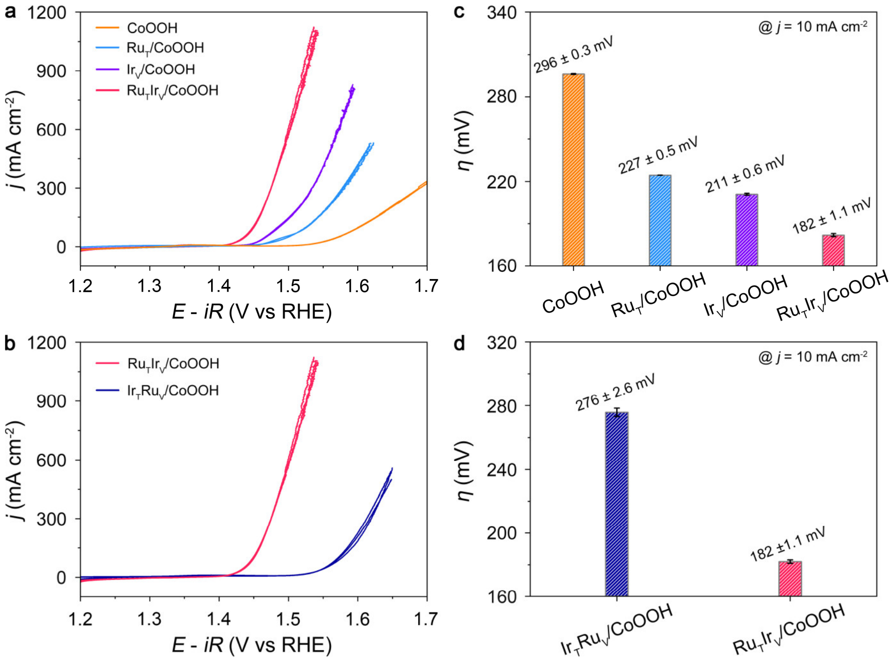

Supplementary Figure 21 | Electrocatalytic performance of the samples towards oxygen evolution in three independent experiments. a. **b**. Polarization curves of samples towards oxygen evolution in 1.0 M KOH electrolyte using Hg/HgO as the reference electrode. **c**. **d**. Overpotentials at a current density of 10 mA cm^{-2} in each test for the samples with error bars. Source data are provided as a Source Data file.

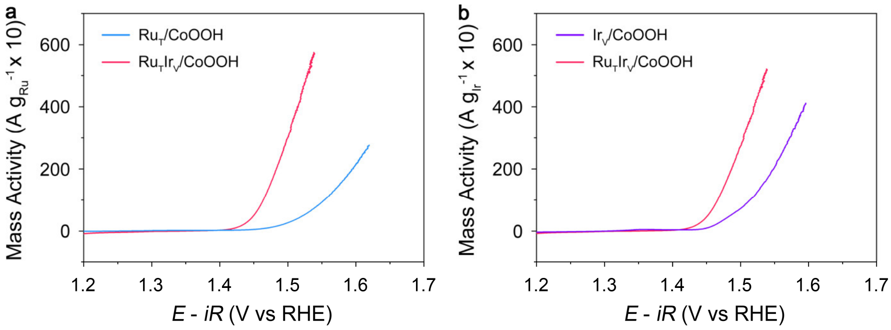

Supplementary Figure 22 | Mass activities of the Ru_T/CoOOH, Ir_V/CoOOH, and Ru_TIr_V/CoOOH. a. Polarization curves of the mass activities against the mass loadings of Ru single atoms on Ru_T/CoOOH and Ru_TIr_V/CoOOH. **b**. Polarization curves of the mass activities against the mass loadings of Ir single atoms on Ir_V/CoOOH and Ru_TIr_V/CoOOH. Source data are provided as a Source Data file.

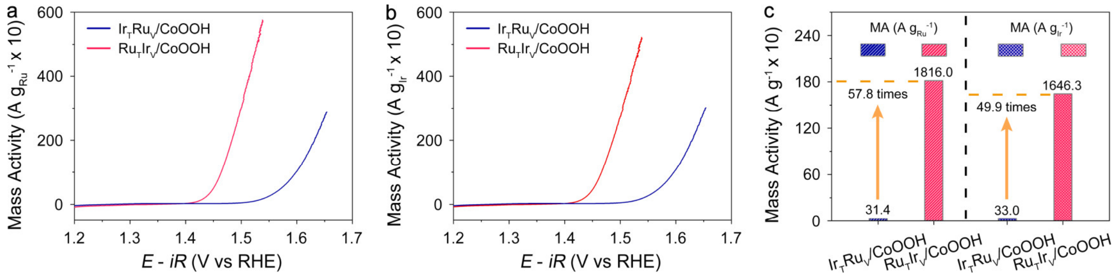

Supplementary Figure 23 | Mass activities of the Ir_TRu_V/CoOOH and Ru_TIr_V/CoOOH. a. b. Polarization curves of the mass activities against the mass loadings of Ru (**a**) and Ir (**b**) single atoms on Ir_TRu_V/CoOOH and Ru_TIr_V/CoOOH. **c**. Mass activities of Ir_TRu_V/CoOOH and Ru_TIr_V/CoOOH against the mass loadings of Ru and Ir single atoms at an overpotential of 250 mV. Source data are provided as a Source Data file.

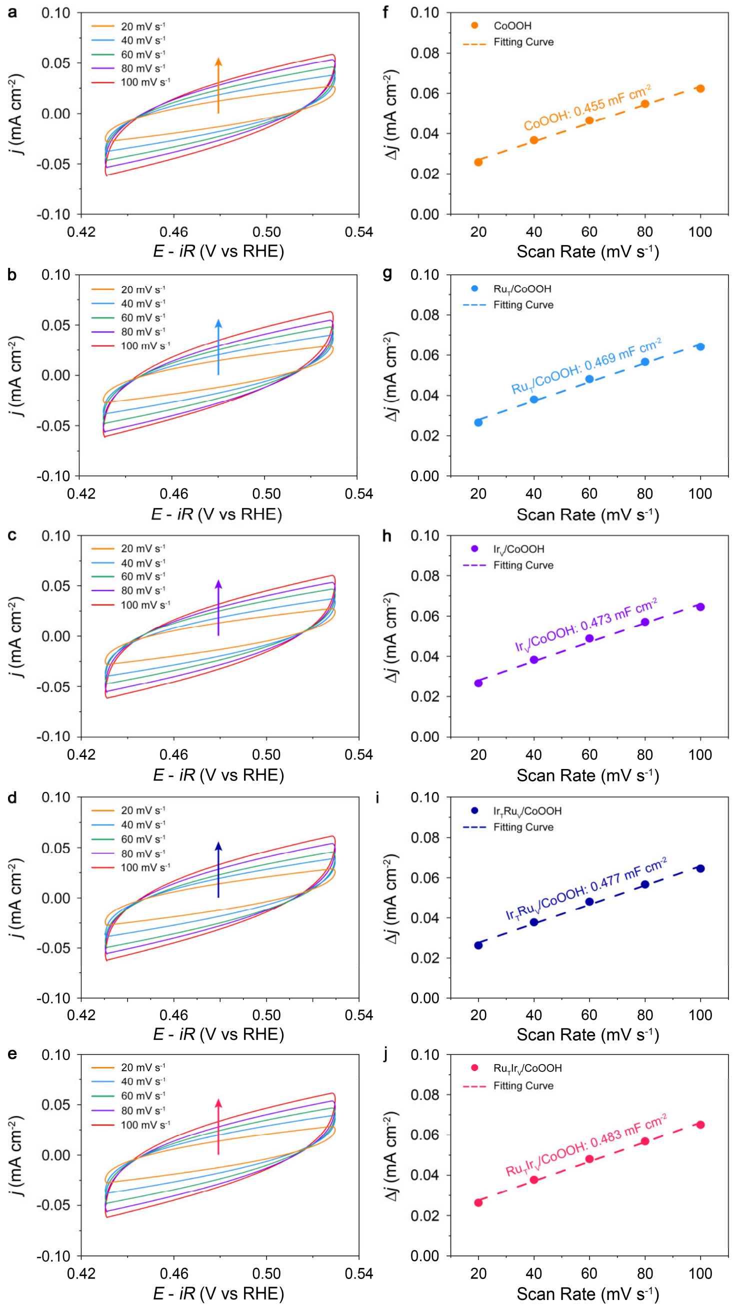

Supplementary Figure 24 | CV curves and charging current density differences of CoOOH, Ru_T/CoOOH, Ir_V/CoOOH, Ir_TRu_V/CoOOH, and Ru_TIr_V/CoOOH. a-e. CV curves of CoOOH (**a**), Ru_T/CoOOH (**b**), Ir_V/CoOOH (**c**), Ir_TRu_V/CoOOH (**d**), and Ru_TIr_V/CoOOH (**e**), respectively. **f-j**. Charging current density differences of CoOOH (**f**), Ru_T/CoOOH (**g**), Ir_V/CoOOH (**h**), Ir_TRu_V/CoOOH (**i**), and Ru_TIr_V/CoOOH (**j**), respectively. Source data are provided as a Source Data file.

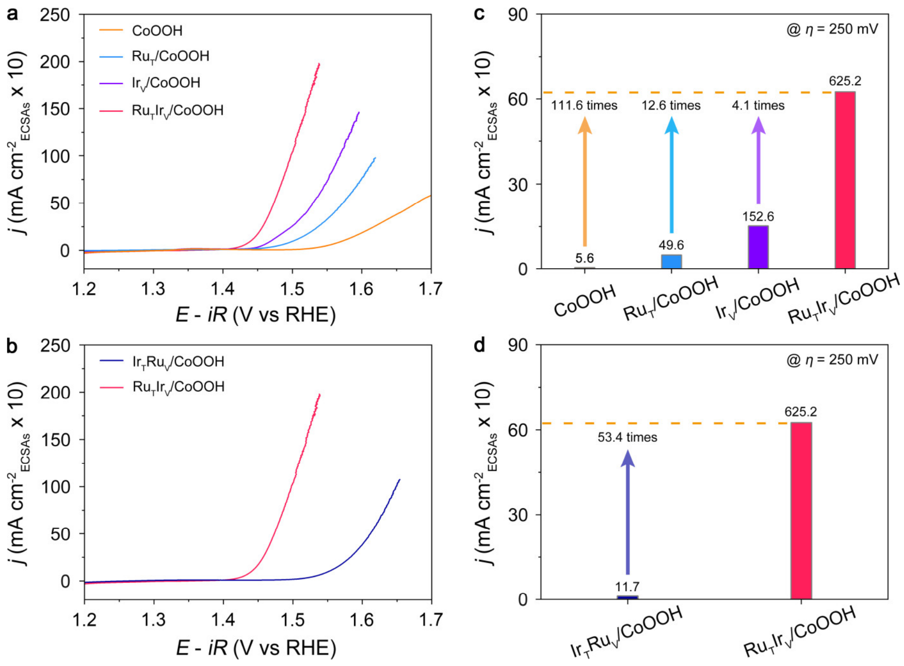

Supplementary Figure 25 | Specific activities of the samples normalized against ECSAs. a. Polarization curves of CoOOH, Ru_T/CoOOH, Ir_V/CoOOH, and Ru_TIr_V/CoOOH towards oxygen evolution in 1.0 M KOH electrolyte against ECSAs. **b**. The specific activities of the samples at an overpotential of 250 mV. **c**. Polarization curves of Ru_TIr_V/CoOOH and Ir_TRu_V/CoOOH towards oxygen evolution in 1.0 M KOH electrolyte against ECSAs. **d**. The specific activities of Ru_TIr_V/CoOOH and Ir_TRu_V/CoOOH at an overpotential of 250 mV. Source data are provided as a Source Data file.

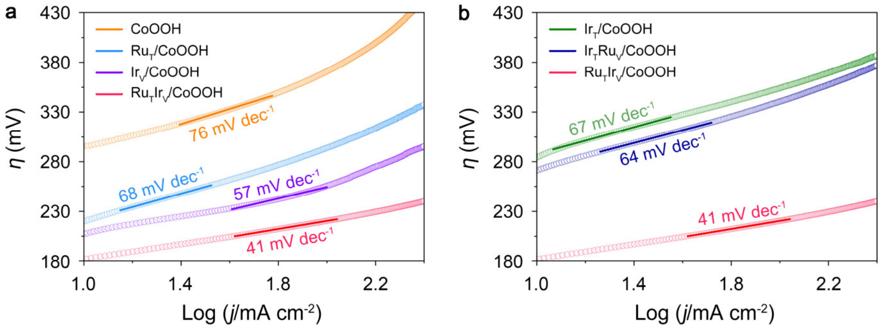

Supplementary Figure 26 | Tafel slopes. a. Tafel slopes of CoOOH, Ru_T/CoOOH, Ir_V/CoOOH, and Ru_TIr_V/CoOOH. **b**. Tafel slopes of Ir_T/CoOOH, Ir_TRu_v/CoOOH, and Ru_TIr_V/CoOOH. Source data are provided as a Source Data file.

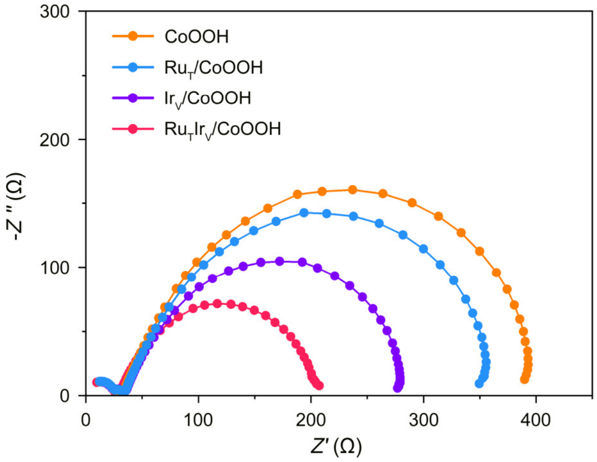

Supplementary Figure 27 | Electrochemical impedance spectra. Electrochemical impedance spectra of CoOOH, Ru_T/CoOOH, Ir_V/CoOOH, and Ru_TIr_V/CoOOH. Source data are provided as a Source Data file.

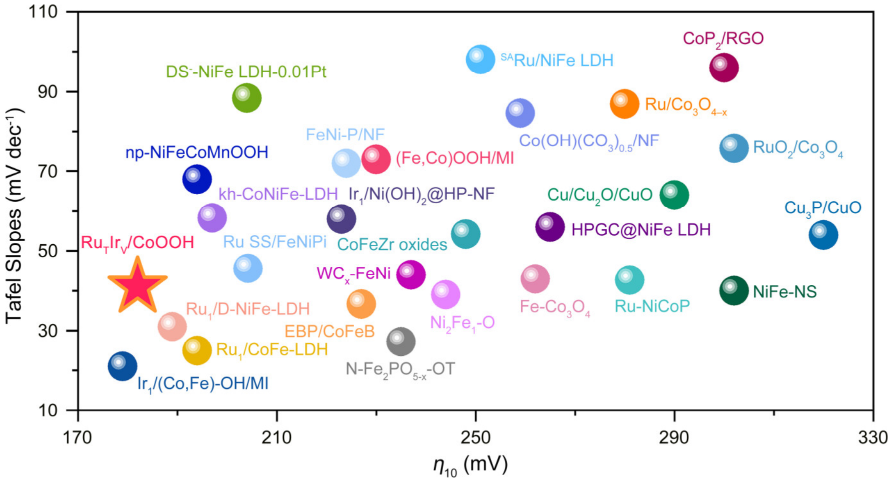

Supplementary Figure 28 | Performance comparison. Comparison of overpotential at a current density of 10 mA cm^{-2} and Tafel slopes for recently reported catalysts in alkaline electrolyte. Source data are provided as a Source Data file.

Supplementary Table 5. Comparison of oxygen evolution performance and Tafel slopes for recently reported catalysts in alkaline electrolyte.

Catalysts	Electrolyte	Overpotential (mV) @ j = 10 mA cm^{-2}	Tafel slopes (mV dec^{-1})	Ref.
**Ru_TIr_V/CoOOH**	**1.0 M KOH**	**180**	**41**	**This work**
Ir_1/(Co,Fe)-OH/MI	1.0 M KOH	179	24	1
Ru_1/D-NiFe-LDH	1.0 M KOH	189	31	2
Ru_1/CoFe-LDH	1.0 M KOH	194	25	3
np-NiFeCoMnOOH	1.0 M KOH	194	67.96	4
kh-CoNiFe-LDH	1.0 M KOH	197	58.3	5
DS^--NiFe LDH-0.01Pt	1.0 M KOH	204	90.3	6
Ru SS/FeNiPi	1.0 M KOH	204.2	45.6	7
$Ir_1/Ni(OH)_2$@HP-NF	1.0 M KOH	223	58	8
FeNi-P/NF	1.0 M KOH	224	72	9
EBP/CoFeB	1.0 M KOH	227	36.7	10
(Fe,Co)OOH/MI	1.0 M KOH	230	73	11
N-Fe_2PO_{5-x}-OT	1.0 M KOH	235	27.2	12
WC_x-FeNi	1.0 M KOH	237	44	13
Ni_2Fe_1-O	1.0 M KOH	244	39	14
CoFeZr oxides	1.0 M KOH	248	54.2	15
^{SA}Ru/NiFe LDH	1.0 M KOH	251	98.1	16
$Co(OH)(CO_3)_{0.5}$/NF	1.0 M KOH	259	84.6	17
Fe-Co_3O_4	1.0 M KOH	262	43	18
HPGC@NiFe	1.0 M KOH	265	56	19
Ru/Co_3O_{4-x}	1.0 M KOH	280	86.9	20
Ru-NiCoP	1.0 M KOH	281	42.7	21
Cu/Cu_2O/CuO	1.0 M NaOH	290	64	22

CoP_2/RGO	1.0 M KOH	300	96	23
NiFe-NS	1.0 M KOH	302	40	24
RuO_2/Co_3O_4	1.0 M KOH	302	75.77	25
Cu_3P/CuO	1.0 M KOH	320	54	26

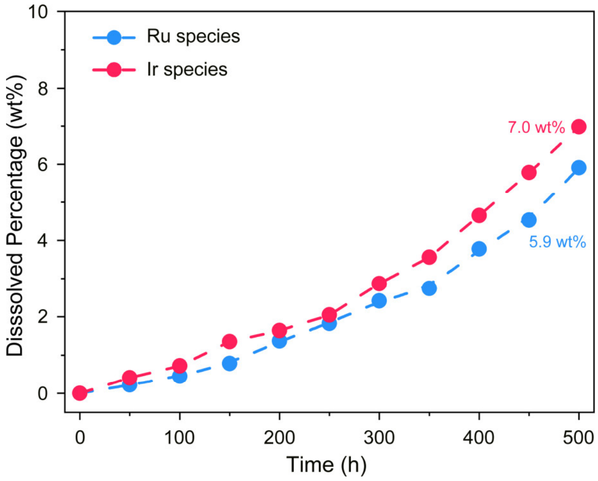

Supplementary Figure 29 | The dissolved percentage of Ru and Ir species during the stability test. The dissolved Ru and Ir species were detected by ICP-MS during the stability test. Source data are provided as a Source Data file.

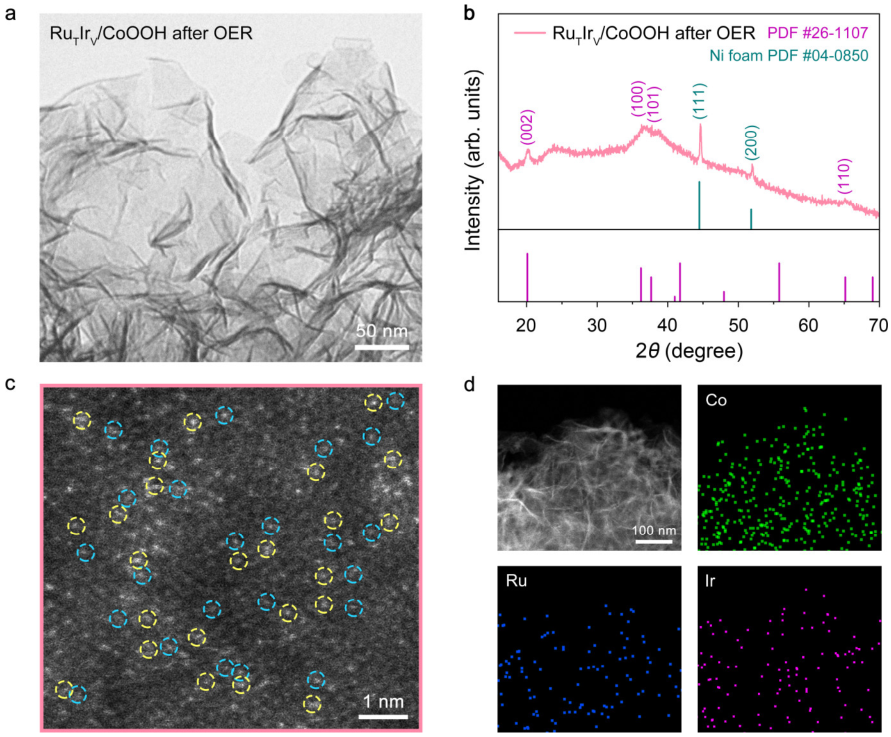

Supplementary Figure 30 | Morphology and structure characterizations of Ru_TIr_V/CoOOH after OER. **a**. TEM image. **b**. XRD pattern. **c**. HAADF-STEM image. **d**. EDX elemental mapping. Source data are provided as a Source Data file.

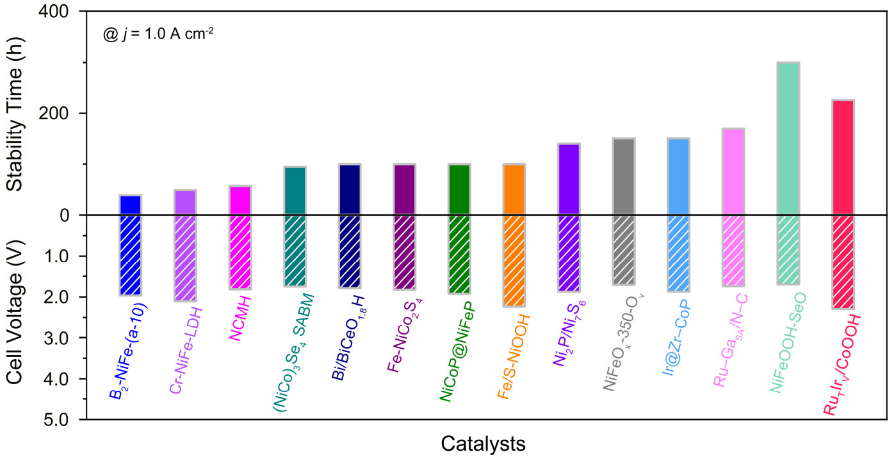

Supplementary Figure 31 | Performance comparison. Comparison of cell voltage and stability time at a current density of 1.0 A cm^{-2} for recently reported catalysts applied in AEM water electrolyzers. Source data are provided as a Source Data file.

Supplementary Table 6. Comparison of cell voltage and stability time at a current density of 1.0 A cm^{-2} for recently reported catalysts applied in AEM water electrolyzers.

Catalysts	Electrolyte	Stability Time (h)	Cell Voltage (V) @ j = 1.0 A cm^{-2}	Ref.
**Ru_TIr_V/CoOOH**	**1.0 M KOH**	**226**	**2.30**	**This work**
B_2-NiFe-(a-10)	1.0 M KOH	40	1.97	27
Cr-NiFe-LDH	1.0 M KOH	50	2.11	28
NCMH	1.0 M KOH	58	1.82	29
$(NiCo)_3Se_4$ SABM	1.0 M KOH	95	1.75	30
$Bi/BiCeO_{1.8}H$	1.0 M KOH	100	1.79	31
Fe-$NiCo_2S_4$	1.0 M KOH	100	1.83	32
NiCoP@NiFeP	1.0 M KOH	100	1.93	33
Fe/S-NiOOH	1.0 M KOH	100	2.24	34
Ni_2P/Ni_7S_6	1.0 M KOH	140	1.88	35
$NiFeO_x$-350-O_v	1.0 M KOH	150	1.71	36
Ir@Zr-CoP	1.0 M KOH	150	1.88	37
Ru-Ga_{SA}/N-C	1.0 M KOH	170	1.74	38
NiFeOOH-SeO	1.0 M KOH	300	1.70	39

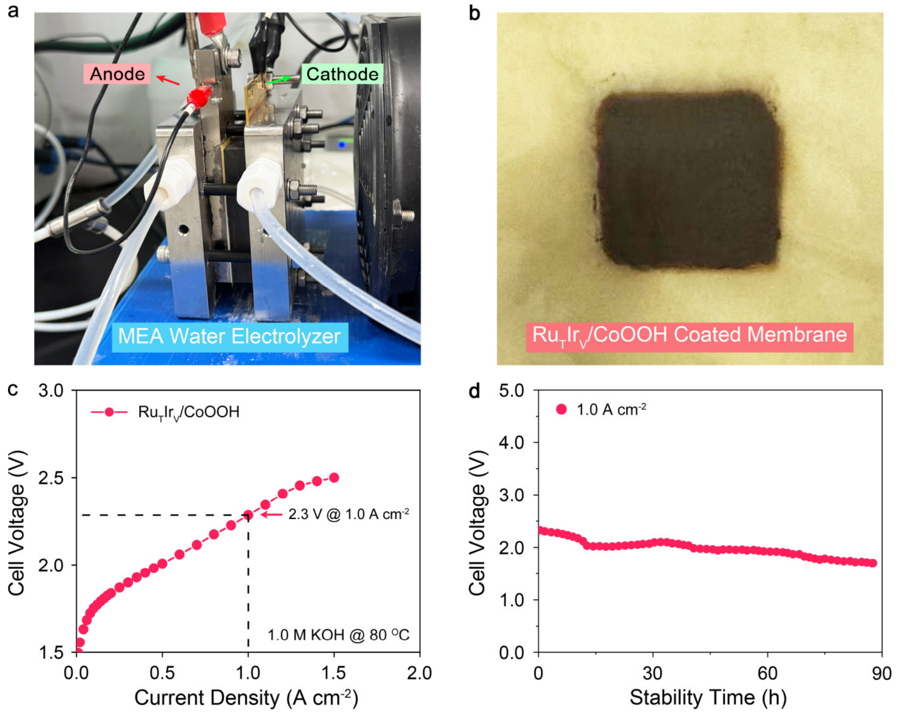

Supplementary Figure 32 | MEA water electrolyzer tests. a. **b**. Optical image of the MEA water electrolyzer (**a**) and the Ru_TIr_V/CoOOH coated membrane (**b**). **c**. Polarization curves of Ru_TIr_V/CoOOH towards OER in the MEA water electrolyzer without *iR*-compensation. **d**. Chronopotentiometry curves of Ru_TIr_V/CoOOH towards OER at a current density of 1.0 A cm^{-2} in the MEA water electrolyzer. Source data are provided as a Source Data file.

a b

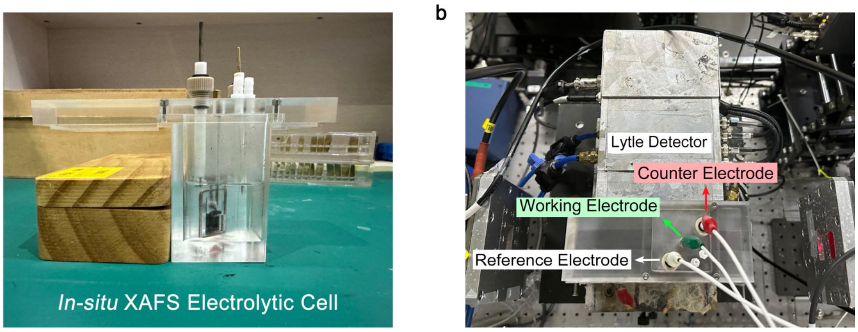

Supplementary Figure 33 | *In-situ* XAFS characterizations. a. Optical image of the *in-situ* XAFS electrolytic cell. **b**. The device for the *in-situ* XAFS measurements.

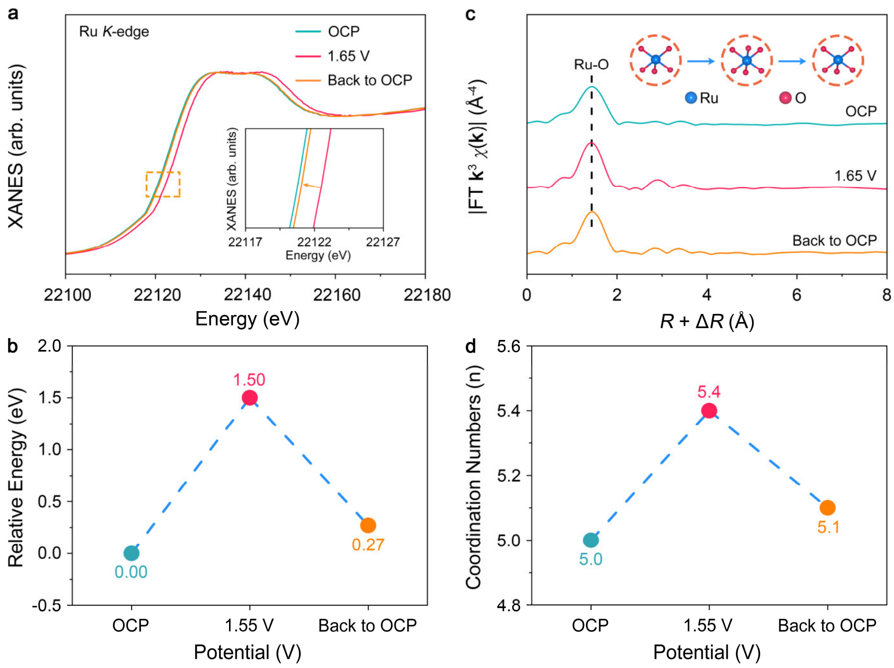

Supplementary Figure 34 | *In-situ* Ru *K*-edge XAFS characterizations. a. *In-situ* Ru *K*-edge XANES of Ru_TIr_V/CoOOH. **b**. The relative energy of absorption edge in Ru *K*-edge XANES under different applied potentials. **c**. Experimental and fitting *in-situ* EXAFS spectra of Ru_TIr_V/CoOOH at the Ru *K*-edge under applied potentials of OCP, 1.65 V, and back to OCP. The experimental and fitting results are indicated as circles and solid lines, respectively. *R* and **k** denoted radial distance and wave vector, respectively. The inset atomic models are the configurations of Ru single atoms during OER. The red and blue spheres represent O and Ru atoms, respectively. **d**. The coordination numbers of Ru single atoms during the *in-situ* XAFS measurements under applied potentials of OCP, 1.65 V, and back to OCP. Source data are provided as a Source Data file.

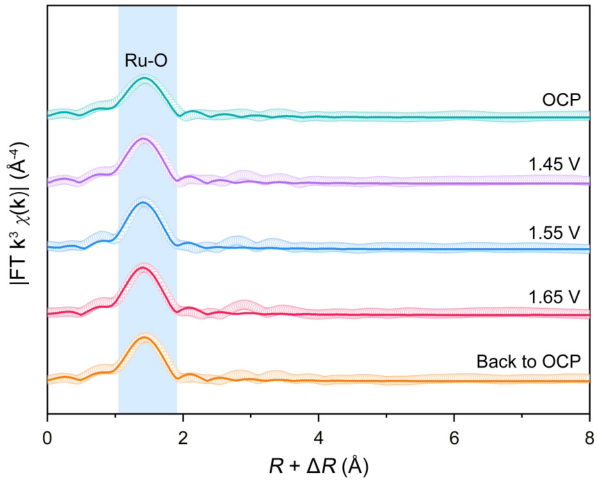

Supplementary Figure 35 | Experimental and fitting *in-situ* EXAFS spectra of Ru_TIr_V/CoOOH at the Ru *K*-edge under applied potentials of OCP, 1.45, 1.55, 1.65 V, and back to OCP. The experimental and fitting results are indicated as circles and solid lines, respectively. *R* and **k** denoted radial distance and wave vector, respectively. Source data are provided as a Source Data file.

Supplementary Table 7 | Fitting results of *in-situ* Ru *K*-edge EXAFS spectra for $Ru_TIr_V/CoOOH$.

Applied voltage	Path	*R* (Å)	*CN*s	σ^2 (10^{-3})	ΔE_0 (eV)	*R*-factor
OCP	Ru-O	2.00 ± 0.02	5.0 ± 1.2	6.4	-2.0	0.02
1.45 V	Ru-O	2.00 ± 0.02	5.1 ± 1.0	5.6	-3.2	0.01
1.55 V	Ru-O	2.00 ± 0.02	5.3 ± 0.8	6.1	-2.6	0.01
1.65 V	Ru-O	2.00 ± 0.01	5.4 ± 0.8	6.0	-2.2	0.01
Back to OCP	Ru-O	2.00 ± 0.02	5.1 ± 0.9	5.9	-2.8	0.01

R, the distance between the absorber and backscatter atoms. The *R* value was phase corrected during fitting process; *CN*s, coordination numbers; σ^2, Debye-Waller factors; ΔE_0, the inner potential correction that accounts for the difference in the inner potential between the sample and the references; S_0^2, the amplitude reduction factor, the S_0^2 for the *in-situ* Ru *K*-edge EXAFS spectra fitting was determined to be 0.89; *R*-factor, the goodness of fit.

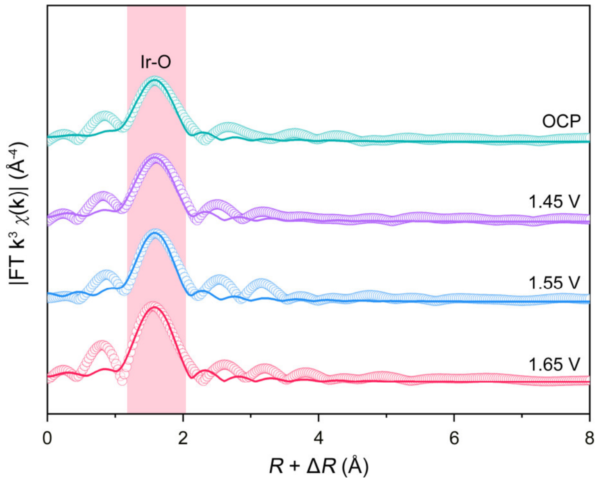

Supplementary Figure 36 | Experimental and fitting *in-situ* EXAFS spectra of Ru_TIr_V/CoOOH at the Ir L_3-edge under applied potentials ranging from OCP to 1.65 V. The experimental and fitting results are indicated as circles and solid lines, respectively. *R* and **k** denoted radial distance and wave vector, respectively. Source data are provided as a Source Data file.

Supplementary Table 8 | Fitting results of *in-situ* Ir L_3-edge EXAFS spectra for Ru_TIr_V/CoOOH.

Applied voltage	Path	*R* (Å)	*CN*s	σ^2 (10^{-3})	ΔE_0 (eV)	*R*-factor
OCP	Ir-O	2.00 ± 0.01	6.0 ± 1.1	9.6	10.0	0.02
1.45 V	Ir-O	2.00 ± 0.01	6.0 ± 1.1	8.7	10.0	0.01
1.55 V	Ir-O	2.00 ± 0.01	6.0 ± 1.1	8.1	10.0	0.02
1.65 V	Ir-O	2.00 ± 0.01	6.0 ± 1.1	6.9	10.0	0.02

R, the distance between the absorber and backscatter atoms. The *R* value was phase corrected during fitting process; *CN*s, coordination numbers; σ^2, Debye-Waller factors; ΔE_0, the inner potential correction that accounts for the difference in the inner potential between the sample and the references; S_0^2, the amplitude reduction factor, the S_0^2 for the *in-situ* Ru *K*-edge EXAFS spectra fitting was determined to be 1.05; *R*-factor, the goodness of fit.

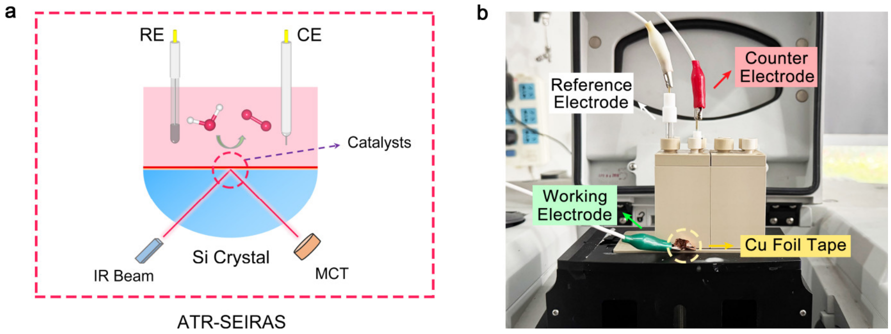

Supplementary Figure 37 | *In-situ* spectroscopic characterizations. a. Schematic diagram of the *in-situ* ATR-SEIRAS measurements. **b**. Optical image of the *in-situ* ATR-SEIRAS measurements.

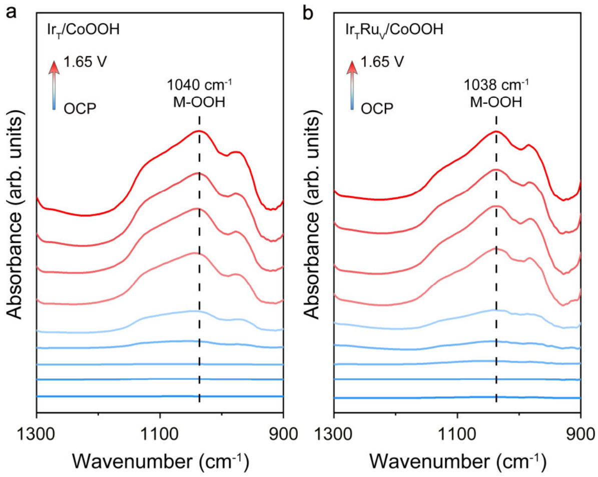

Supplementary Figure 38 | *In-situ* spectroscopic analysis. a. **b**. *In-situ* ATR-SEIRAS of Ir_T/CoOOH (**a**) and Ir_TRu_V/CoOOH (**b**). Source data are provided as a Source Data file.

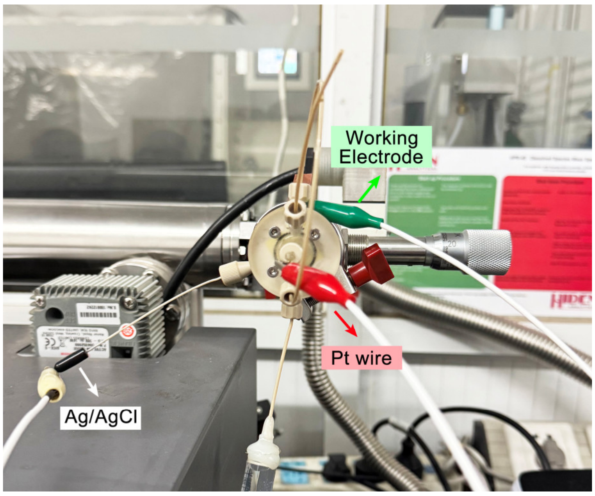

Supplementary Figure 39 | *In-situ* ^{18}O isotope-labeling DEMS characterizations. Optical image of the *in-situ* ^{18}O isotope-labeling DEMS experiments.

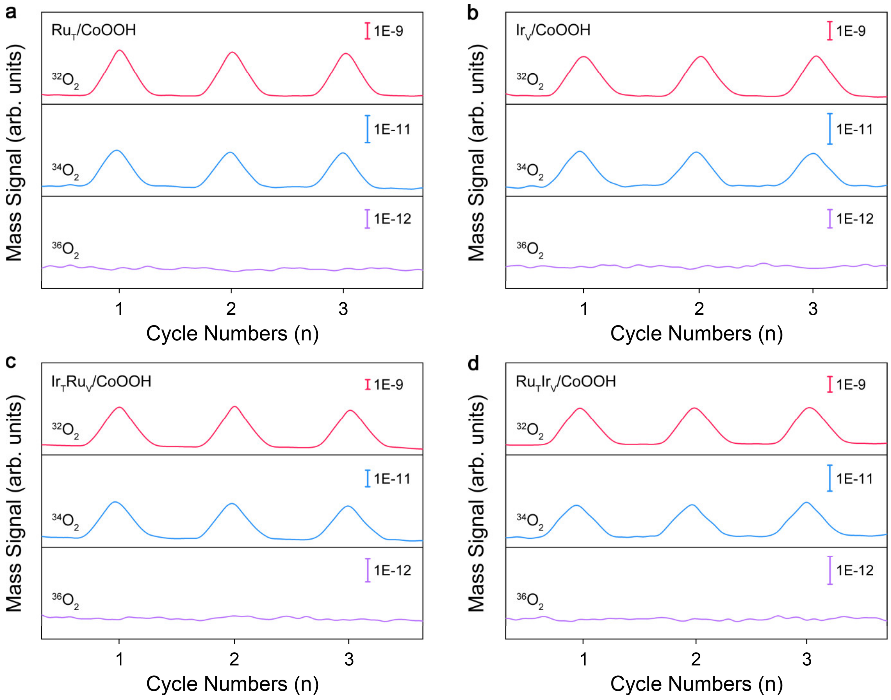

Supplementary Figure 40 | *In-situ* ^{18}O isotope-labeling DEMS measurements. a-d. *In-situ* DEMS signals of $^{36}O_2$, $^{34}O_2$, and $^{32}O_2$ for Ru_T/CoOOH (**a**), Ir_V/CoOOH (**b**), Ir_TRu_V/CoOOH (**c**), and Ru_TIr_V/CoOOH (**d**). Source data are provided as a Source Data file.

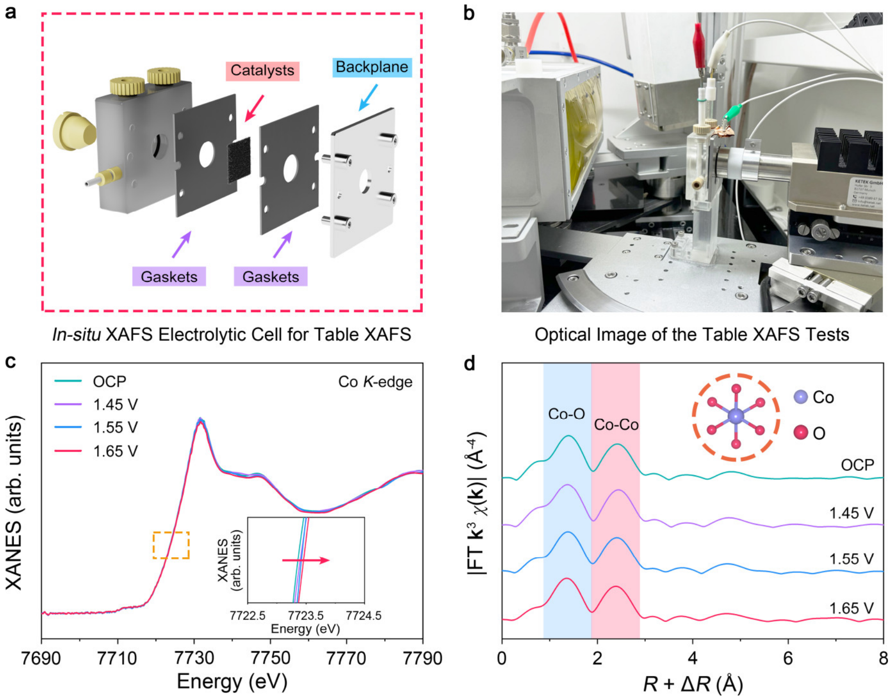

Supplementary Figure 41 | *In-situ* spectroscopic characterizations. a. Schematic diagram of the *in-situ* XAFS electrolytic cell. **b**. Optical image of the *in-situ* Co *K*-edge XAFS tests. **c**. **d**. *In-situ* Co *K*-edge XANES (**c**) and EXAFS (**d**) spectra of Ru_TIr_V/CoOOH under applied potentials ranging from OCP to 1.65 V. *R* and **k** denoted radial distance and wave vector, respectively. Source data are provided as a Source Data file.

Supplementary Table 9. Gibbs free energies (Δ*G*) of OER intermediates adsorbed on the Ru sites at the surface of Ru_T/CoOOH and Ru_TIr_V/CoOOH, respectively.

Samples	ΔG_{*OH} (eV)	ΔG_{*O} (eV)	ΔG_{*OOH} (eV)	Theoretical overpotential (V)
Ru_T/CoOOH	0.63	1.22	4.00	1.55
Ru_TIr_V/CoOOH	0.45	1.06	2.76	0.93

Supplementary References

1. Zhao, J. et al. Out-of-plane coordination of iridium single atoms with organic molecules and cobalt-iron hydroxides to boost oxygen evolution reaction. *Nat. Nanotechnol.* **20**, 57-66 (2025).
2. Zhai, P. et al. Engineering single-atomic ruthenium catalytic sites on defective nickel-iron layered double hydroxide for overall water splitting. *Nat Commun.* **12**, 4587 (2021).
3. Mu, X. et al. Breaking the symmetry of single-atom catalysts enables an extremely low energy barrier and high stability for large-current-density water splitting. *Energy Environ. Sci.* **15**, 4048-4057 (2022).
4. Zhang, Y. et al. Boosting the oxygen evolution of high-entropy (oxy)hydroxide epitaxially grown on high entropy alloy by lattice oxygen activation. *Appl. Catal. B: Environ.* **341**, 123331 (2024).
5. Wang, P. et al. High-density defects in ordered macroporous-mesoporous CoNiFe-LDHs for efficient and robust oxygen evolution reaction. *Chem Catal.* **3**, 100497 (2023).
6. Zheng, Z. et al. Anchoring active sites by Pt_2FeNi alloy nanoparticles on NiFe layered double hydroxides for efficient electrocatalytic oxygen evolution reaction. *Energy Environ. Mater.* **5**, 270-277 (2022).
7. Lu, S-Y. et al. Synthetic tuning stabilizes a high-valence Ru single site for efficient electrolysis. *Nat. Synth.* **3**, 576-585 (2024).
8. Jia, C. et al. Ir single atoms modified $Ni(OH)_2$ nanosheets on hierarchical porous nickel foam for efficient oxygen evolution. *Nano Res.* **15**, 10014-10020 (2022).
9. Yan, Q. et al. Self-supported FeNi-P nanosheets with thin amorphous layers for efficient electrocatalytic water splitting. *ACS Sustain. Chem. Eng.* **6**, 9640-9648 (2018).
10. Chen, H. et al. 2D heterostructure of amorphous CoFeB coating black phosphorus nanosheets with optimal oxygen intermediate absorption for improved electrocatalytic water oxidation. *ACS Nano* **15**, 12418-12428 (2021).
11. Huang, W. et al. Ligand modulation of active sites to promote electrocatalytic oxygen evolution. *Adv. Mater.* **34**, 2200270 (2022).
12. Wu, Y. et al. Orienting active crystal planes of new class lacunaris Fe_2PO_5 polyhedrons for robust water oxidation in alkaline and neutral media. *Adv. Funct. Mater.* **28**, 1801397 (2018).
13. Li, S. et al. Oxygen-evolving catalytic atoms on metal carbides. *Nat. Mater.* **20**, 1240-1247

(2021).
14. Dong, C. et al. Eutectic-derived mesoporous Ni-Fe-O nanowire network catalyzing oxygen evolution and overall water splitting. *Adv. Energy Mater.* **8**, 1701347 (2018).
15. Huang, L. et al. Zirconium-regulation-induced bifunctionality in 3D cobalt-iron oxide nanosheets for overall water splitting. *Adv. Mater.* **31**, 1901439 (2019).
16. Yang, Y. et al. Enhancing water oxidation of Ru single atoms via oxygen-coordination bonding with NiFe layered double hydroxide. *ACS Catal.* **13**, 2771-2779 (2023).
17. Liang, R. et al. Understanding the anion effect of basic cobalt salts for the electrocatalytic oxygen evolution reaction. *ACS Catal.* **13**, 8821-8829 (2023).
18. Zhang, S. L. et al. Metal atom-doped Co_3O_4 hierarchical nanoplates for electrocatalytic oxygen evolution. *Adv. Mater.* **32**, 2002235 (2020).
19. Ni, Y. et al. Construction of hierarchically porous graphitized carbon-supported NiFe layered double hydroxides with a core-shell structure as an enhanced electrocatalyst for the oxygen evolution reaction. *Nanoscale* **9**, 11596-11604 (2017).
20. Yuan C-Z. et al. In situ immobilizing atomically dispersed Ru on oxygen-defective Co_3O_4 for efficient oxygen evolution. *ACS Catal.* **13**, 2462-2471 (2023).
21. Kim J. et al. Facile one-step synthesis of Ru doped NiCoP nanoparticles as highly efficient electrocatalysts for oxygen evolution reaction. *Chem-Asian J.* **16**, 3630-3635 (2021).
22. Huan, T. N. et al. A dendritic nanostructured copper oxide electrocatalyst for the oxygen evolution reaction. *Angew. Chem. Int. Ed.* **56**, 4792-4796 (2017).
23. Wang, J.; Yang, W.; Liu, J. et al. CoP_2 nanoparticles on reduced graphene oxide sheets as a super-efficient bifunctional electrocatalyst for full water splitting. *J. Mater. Chem. A* **4**, 4686-4690 (2016).
24. Song, F. & Hu X. Exfoliation of layered double hydroxides for enhanced oxygen evolution catalysis. *Nat. Commun.* **5**, 4477 (2014).
25. Guo, B. et al. RuO_2/Co_3O_4 nanocubes based on Ru ions impregnation into prussian blue precursor for oxygen evolution. *Int. J. Hydrogen Energy* **45**, 9575-9582 (2020).
26. Han, A. et al. Crystalline copper phosphide nanosheets as an efficient Janus catalyst for overall water splitting. *ACS Appl. Mater. Interfaces* **9**, 2240-2248 (2020).
27. Ma, Y. et al. Short-time potentiostatic assisted borate to induce the generation of ultrathin NiFe LDH active phase for industrial-level water oxidation. *Chem. Eng. J.* **490**, 151490

(2024).

28. Wang, M. H. et al. Operando high-valence Cr-modified NiFe hydroxides for water oxidation. *Small* **18**, 2200303 (2022).
29. Karmakar, A. et al. Surface hydroxyl group-enriched nickel cobalt molybdate hydrate for improved oxygen evolution activity in an anion exchange membrane water electrolyzer. *Appl. Catal. B* **328**, 122504 (2023).
30. Abed. J. et al. In situ formation of nano Ni-Co oxyhydroxide enables water oxidation electrocatalysts durable at high current densities. *Adv. Mater.* **33**, 2103812 (2021).
31. Jo. S. et al. Stabilization of lattice oxygen evolution reactions in oxophilic Ce-mediated Bi/$BiCeO_{1.8}H$ electrocatalysts for efficient anion exchange membrane water electrolyzers. *Adv. Mater.* **36**, 2314211 (2024).
32. Wang. F-L. et al. Trojan strategy assisted phase-pure Fe-$NiCo_2S_4$ for industrial anion-exchange membrane water electrolyzer. *Appl. Catal. B Environ.* **331**, 122660 (2023).
33. Zhao. Y. et al. Homologous NiCoP@NiFeP heterojunction array achieving high-current hydrogen evolution for alkaline anion exchange membrane electrolyzers. *J. Mater. Chem. A* **10**, 10209-10218 (2022).
34. Wang. F-L. et al. In situ electrochemical rapid induction of highly active γ-NiOOH species for industrial anion exchange membrane water electrolyzer. *Small* **20**, 2310064 (2024).
35. Wang. F-L. et al. Porous heterojunction of Ni_2P/Ni_7S_6 with high crystalline phase and superior conductivity for industrial anion exchange membrane water electrolysis. *Appl. Catal. B Environ.* **330**, 122633 (2023).
36. Lee. H. et al. A chalcogenide-derived $NiFe_2O_4$ as highly efficient and stable anode for anion exchange membrane water electrolysis. *Chem. Eur. J.* e202403198 (2024).
37. Ngo. Q. P. et al. Unveiling the synergistic effect of atomic iridium modulated zirconium-doped pure phase cobalt phosphide for robust anion-exchange membrane water electrolyzer. *Adv. Energy Mater.* **13**, 2301841 (2023).
38. Zhou. C. et al. Oxophilic gallium single atoms bridged ruthenium clusters for practical anion-exchange membrane electrolyzer. *Nat. Commun.* **15**, 6741 (2024).
39. Zhang. Y. et al. Selenate oxyanion-intercalated NiFeOOH for stable water oxidation via lattice oxygen oxidation mechanism. *J. Energy Chem.* **101**, 676-684 (2025).
