# Supplementary material for: Site-specific synergy in heterogeneous single atoms for efficient oxygen evolution
Source: Nat Commun. 2025 Mar 15;16:2573. doi: 10.1038/s41467-025-57864-w (PMC11910543; doi:10.1038/s41467-025-57864-w)
Supplement: Supplementary file 2 — Description of Additional Supplementary Files [file 41467_2025_57864_MOESM2_ESM.pdf]

Description of Additional Supplementary Files

Supplementary Data 1. Optimized structural models of CoOOH, Ru_T/CoOOH, and Ru_TIr_V/CoOOH.
