# Supplementary material for: Site-specific synergy in heterogeneous single atoms for efficient oxygen evolution
Source: Nat Commun. 2025 Mar 15;16:2573. doi: 10.1038/s41467-025-57864-w (PMC11910543; doi:10.1038/s41467-025-57864-w)
Supplement: Supplementary file 3 — Supplementary Data 1 [file 41467_2025_57864_MOESM3_ESM.zip › Supplementary Data 1/The legend of the Supplementary Data 1.doc]

**Supplementary Data 1.** Optimized structural models of CoOOH, Ru_T_/CoOOH, and Ru_T_Ir_V_/CoOOH.
